# Application of Rapid Fluorescence Lifetime Imaging Microscopy (RapidFLIM) to Examine Dynamics of Nanoparticle Uptake in Live Cells

**DOI:** 10.3390/cells11040642

**Published:** 2022-02-12

**Authors:** Aria Ahmed-Cox, Alexander M. Macmillan, Elvis Pandzic, Renee M. Whan, Maria Kavallaris

**Affiliations:** 1 Children’s Cancer Institute, Lowy Cancer Research Center, UNSW Sydney, Randwick, NSW 2031, Australia; aahmed-cox@ccia.org.au; 2 ARC Center of Excellence in Convergent Bio-Nano Science and Technology, Australian Center for NanoMedicine, UNSW Sydney, Sydney, NSW 2031, Australia; 3 School of Women and Children’s Health, Faculty of Medicine and Health, UNSW Sydney, Sydney, NSW 2031, Australia; 4 Katharina Gaus Light Microscopy Facility, Mark Wainwright Analytical Center, UNSW Sydney, Sydney, NSW 2031, Australia; alex.macmillan@unsw.edu.au (A.M.M.); e.pandzic@unsw.edu.au (E.P.); r.whan@unsw.edu.au (R.M.W.)

**Keywords:** fluorescent lifetime, nanoparticle uptake, tracking, live cell imaging

## Abstract

A key challenge in nanomedicine stems from the continued need for a systematic understanding of the delivery of nanoparticles in live cells. Complexities in delivery are often influenced by the biophysical characteristics of nanoparticles, where even subtle changes to nanoparticle designs can alter cellular uptake, transport and activity. Close examination of these processes, especially with imaging, offers important insights that can aid in future nanoparticle design or translation. Rapid fluorescence lifetime imaging microscopy (RapidFLIM) is a potentially valuable technology for examining intracellular mechanisms of nanoparticle delivery by directly correlating visual data with changes in the biological environment. To date, applications for this technology in nanoparticle research have not been explored. A PicoQuant RapidFLIM system was used together with commercial silica nanoparticles to follow particle uptake in glioblastoma cells. Importantly, RapidFLIM imaging showed significantly improved image acquisition speeds over traditional FLIM, which enabled the tracking of nanoparticle uptake into subcellular compartments. We determined mean lifetime changes and used this to delineate significant changes in nanoparticle lifetimes (>0.39 ns), which showed clustering of these tracks proximal to both extracellular and nuclear membrane boundaries. These findings demonstrate the ability of RapidFLIM to track, localize and quantify changes in single nanoparticle fluorescence lifetimes and highlight RapidFLIM as a valuable tool for multiparameter visualization and analysis of nanoparticle molecular dynamics in live cells.

## 1. Introduction

The effective delivery of novel drugs remains a major challenge for therapeutics in the 21st century. Therapeutic nanoparticles, defined as drug delivery vehicles on the nanoscale size, are a rapidly advancing area of interest that have shown potential to revolutionise therapies in cancer and other diseases [1,2]. However, a key challenge in developing these nanoparticles for clinical use stems from limitations in our understanding of their delivery, namely, how the biophysical characteristics of nanoparticles influence their cellular uptake and transport [3,4,5].

Recently, new imaging technologies have been developed which can assist not only in visualizing nanoparticle uptake by living cells, but in acquiring quantitative biological information during image acquisition. Fluorescence lifetime imaging microscopy (FLIM) is one such technology. FLIM measures the lifetime of a fluorophore: the time it spends in the excited state before returning to the ground state and emitting light [6]. Unlike fluorescence intensity, fluorescence lifetimes are independent of fluorophore concentration, and can distinguish populations of fluorophores in different biochemical environments (pH, temperature, protein concentration) [7,8,9]. FLIM has been applied in studies of drug delivery, including intracellular doxorubicin release, adding a new dimension of intracellular information, which was not previously available from biological data [10,11,12]. Other studies have been interested in visualizing nanoparticle uptake and subcellular trafficking, particularly for environmentally responsive nanoparticles, which can release their drug cargo in response to a change in environment [13,14,15].

Whilst there are several approaches to measuring fluorescence lifetimes including time-gating, pulse-sampling and frequency domain techniques, Time Correlated Single Photon Counting (TCSPC) provides the highest accuracy of lifetime estimation, particularly for complex (multiexponential) samples, with a good signal-to-noise ratio irrespective of photon recovery [16,17]. Unfortunately, a major shortfall of FLIM by TCSPC is the time following the arrival of the first photon where the detector is off (“dead”) as it processes and stores information which is necessary to compute photon arrival times. Even optimised FLIM systems report photon dead times of 50 nanoseconds per photon [18]. To prevent the phenomenon of photon pileup, where high photon counts and long dead times result in a skewing of the fluorescence decay, it is therefore necessary to attenuate photon counts [6]. In practice, the count rate needs to be <10% of the laser repetition rate. Ultimately, this limits the speed of TCSPC FLIM, and means that, to accurately acquire enough photons per pixel for a single frame, approximate imaging times are in the order of minutes [18,19]. This is generally considered too slow to capture mechanisms and cellular processes that occur at a rapid pace, including vesicle and organelle trafficking, limiting the use of FLIM in nanoparticle uptake studies [18,20].

Rapid fluorescence lifetime imaging microscopy (RapidFLIM) was recently developed, with improved hybrid detectors and timing electronics that reduce the overall dead time to single nanoseconds [18]. Having previously been limited by delayed temporal resolution, the advancements of the RapidFLIM system now enable the simultaneous acquisition of lifetimes, spatial information and intensity with significantly higher frame rates than its predecessor [7,8,21]. Given that effective nanoparticle delivery often depends on intracellular trafficking mechanisms, RapidFLIM has the potential to simultaneously capture this uptake and correlate visual data with changes in the biological environment. To date, applications for this technology to study nanoparticle uptake have not been explored. Using a RapidFLIM system and commercial silica nanoparticles, we investigated nanoparticle uptake in cancer cells using RapidFLIM. We hereby establish novel methodologies for the biological characterization and simultaneous multiparameter visualization of nanoparticle uptake and demonstrate the ability of RapidFLIM to track, localize and quantify changes in single particle lifetimes on a nanosecond timescale. This highlights the potential of RapidFLIM as a valuable tool for visualizing and quantifying nanoparticle dynamics in living cells for improved characterization of nanoparticle uptake in cancer research.

## 2. Materials and Methods

### 2.1. Cell Culture and Nanoparticles

U87 glioblastoma (ATCC HTB-14) cells were provided by the Children’s Cancer Institute Cell Bank (Sydney, Australia), and cultured in Dulbecco’s Modified Eagle Medium (DMEM) (Sigma-Aldrich, Castle Hill, Australia) with 10% fetal bovine serum (FBS) (Life Technologies, Mulgrave, Australia). Cells were screened routinely and found to be free of mycoplasma. Sicastar^®^-F red fluorescent silica nanoparticles were purchased from Micromod Partikeltechnologie GmbH (Rostock, Germany) in sizes of 30 nm and 100 nm, which represent what is typically considered the optimal size range for nanoparticle development for clinical application [22,23]. These particles were pre-loaded with Rhodamine B with peak excitation and emission reported to be 569/585 nm, respectively. The specifications and characterizations of these materials per the manufacturer are available on request.

### 2.2. Sample Preparation for 2D Lifetime Imaging of Nanoparticles in Glioblastoma

Sterile, glass-bottom Fluorodish (23–35 mm) cell culture dishes (Worldwide Precision Instruments, Coherent Scientific, Thebarton, Australia) were coated with 500 mL of 0.1 mg/mL rat tail Collagen 1 (Gibco, Thermo Fisher Scientific, Scoresby, Australia) in 1X PBS (Sigma-Aldrich) for 30 min to aid cell attachment to glass, prior to cell seeding. Dishes were rinsed with 1X PBS and glioblastoma (U87) cells were subsequently seeded at 1 × 10^5^ cells for overnight incubation or 5 × 10^4^ cells in 1 mL media for 48-h incubation. Sicastar silica nanoparticles (20 μg/mL of either 30 nm or 100 nm in diameter) were then added to the dishes in preparation for imaging.

### 2.3. Acquisition of Nanoparticle Fluorescence Lifetimes Using RapidFLIM

Fluorodishes were mounted on a PicoQuant Microtime 200 stage (PicoQuant, GmBh, Berlin, Germany), with 1.4 NA 100× objective with Immersion oil type LDF (Cargille Labs, Cedar Grove, NJ, USA) and incubated (37 °C, 5% CO_2_). After equilibration for 1 h, FLIM data was acquired at 510 nm with a laser repetition rate of 40 MHz, minimum 512 × 512-pixel grid and 10 microsecond dwell time per pixel. Frame acquisition was adjusted for an extended time-course (50 frames, every 30 min for 12 h) or rapid acquisition (max of 300 frames, at 2–4 h intervals). Timing electronics were provided by TimeHarp 260 NANO (TH260NANO). Photons were detected by PMA Hybrid detection (PicoQuant). The combination of TH260NANO and PMA Hybrid detection provided the low deadtime solution of RapidFLIM [18].

### 2.4. Phasor Analysis

Phasor analysis was conducted as previously described [24,25,26,27]. In brief, a fluorescence decay at each pixel of the image was measured, Fourier transformed and then plotted onto the universal circle of the phasor plot using the sine and cosine transform of these decays. Each pixel was then mapped within the phasor plot according to its average lifetime value, defined in Equations (1) and (2)

(1) $$S(w)=\frac{\int_{0}^{\infty }I\left(t\right)\sin\left(\omega t\right)dt}{\int_{0}^{\infty }I\left(t\right)dt}$$

(2) $$G(w)=\frac{\int_{0}^{\infty }I\left(t\right)\cos\left(\omega t\right)dt}{\int_{0}^{\infty }I\left(t\right)dt}\;$$

where *G*(*ω*) and *S*(*ω*) were the X and Y coordinates of the phasor transforms. *ω* was the angular repetition frequency (*2πf*) of the excitation source where *f* was the laser repetition rate [24,26]. Phasors were plotted as a two-dimensional phasor plot with the origin in the (0,0) point. 

### 2.5. Temporal Tracking Analysis

For lifetime tracking analysis, frames were exported and unbinned as TIFF files where Channel 1 contained the intensity data and Channel 2 contained the lifetime data per pixel. These were combined as multi-TIFFs in MATLAB (R2020b, Mathworks, Natick, MA, USA) and saved for analysis in DiaTrack (3.05 PRO, Semasopht, Sydney, Australia) [28]. Within DiaTrack pre-processing, the background of the image was subtracted, and data filtered by 0.75–1 pixel and particles generated with an activated high precision mode (set at 0.85–1.5 to match the size of the particles). Dim particles were removed (750–1500) and particle tracks produced. For 30 nm nanoparticle controls embedded in 3% agarose, a maximum jump size between frames was set at 21 pixels, accounting for the greater mean square displacement (MSD) of particles in agarose solution (estimated pore size of less than 50 nm [29]). For biological samples, the maximum jump size was set to 5 pixels to account for increased concentrations of particles within cells, and a reduced MSD expected to be caused by caged diffusion of nanoparticles in intracellular environments. Tracks were saved and imported back into MATLAB to extract the frame-by-frame lifetime per trajectory. Here we considered only tracks measured across 10 or more frames, with a maximum radius of 3 pixels for neighboring features. For thresholding of significant tracks, we first smoothed biological tracks using a moving median of three frames to reduce the likelihood that the lifetime range may be skewed by a single frame shift in lifetime. We then considered tracks where the lifetime range across the length of the trajectory was greater than the mean lifetime variation within agarose-embedded controls.

## 3. Results

### 3.1. RapidFLIM Demonstrates Faster Photon Detection Rates to Resolve Fluorescence Lifetimes

**Figure 1:**
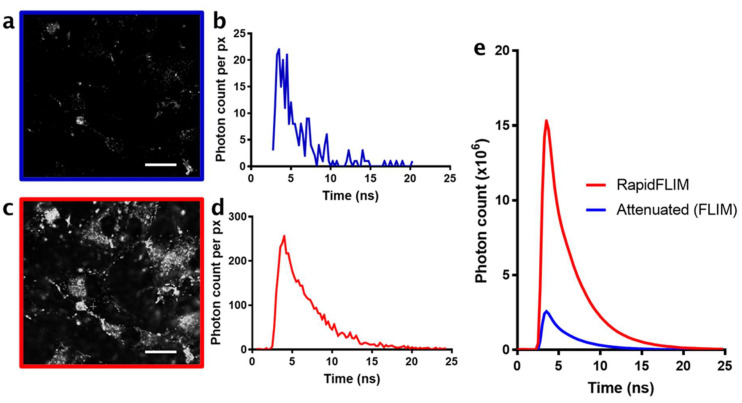
Comparison of photon counts using RapidFLIM versus the attenuation necessary for FLIM detector dead time recovery. (**a**) Attenuated (FLIM) greyscale image of U87 glioblastoma cells following silica nanoparticle (SiNP, 30 nm) addition after 12 h (**b**) Attenuated (FLIM) representative photon counts per pixel over 25 ns, the repetition rate of the laser. (**c**) RapidFLIM greyscale image of U87 glioblastoma cells following silica nanoparticle (SiNP, 30 nm) addition after 12 h, using the same field of view with (**d**) representative photon counts per pixel over 25 ns. (**e**) Overall decays across each frame of attenuated (FLIM, blue line) compared to RapidFLIM acquisition over time. Scale bars, 20 µm.

Herein, we investigated the use of RapidFLIM to improve the multimodal acquisition of nanoparticle cellular uptake to better understand the effects of the biophysical properties of nanoparticle size on uptake and cellular localization. Initially, we established the benefits of RapidFLIM over a traditional FLIM system, by calibrating each system for the rapid lifetime acquisition of nanoparticle uptake. This was conducted using commercial 30 nm silica particles in glioblastoma (U87) cells, at speeds necessary to capture cellular trafficking mechanisms, set here at 100 frames per 30 s acquisition. To emulate a standard FLIM system, detector settings were attenuated to a photon detection rate below 10% of the operating repetition rate of the laser; in this case, four million counts per second (4 Mcps) at 40 MHz, to prevent photon pileup. This demonstrated the challenge of traditional FLIM systems for the acquisition of dynamic processes in biological samples, due to the high number of photons required to accurately determine lifetime (Figure 1a,b). The long pixel dwell time required to collect sufficient photons was typically in the order of milliseconds. This could be a flow-on effect of the long dead times, which required low operational count rates, meaning that most pulses had a high probability of resulting in zero photons collected. Following acquisition, settings were subsequently adjusted for the rapid acquisition of photons permitted by the RapidFLIM timing electronics, and detection applied to the same field of view over the same time frame. In contrast to FLIM, RapidFLIM enabled quality acquisition with detectable biological features (cell protrusions, nuclei; Figure 1c) and up to 10-fold greater photon counts per pixel (Figure 1d) with no photon pileup. These improvements to the hybrid detector demonstrated that the images acquired with RapidFLIM can capture time-tagged acquisition with sustained photon count rates up to 40 Mcps at 40 MHz. Cumulatively, this improved total photon counts across the image by more than 10-fold in RapidFLIM compared to standard FLIM settings (Figure 1e). As a result, in a 30 s time-lapse, we were able to acquire a high-contrast image of intracellular nanoparticle uptake, and high enough photon counts to permit in-depth, downstream analysis.

### 3.2. Particle Size Affected Silica Nanoparticle Lifetimes Acquired during Cellular Uptake

**Figure 2:**
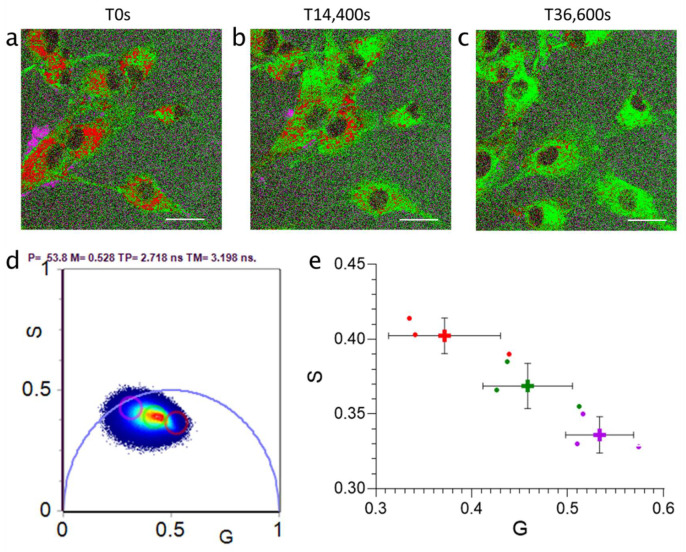
Time-lapse imaging of 30 nm silica nanoparticles (SiNP) uptake in U87 glioblastoma cells captured over 10 h (36,600 s) following SiNP addition. Representative phasor overlay for 30 nm SiNP at (**a**) time 0, (**b**) 4 h and (**c**) 10 h following SiNP addition, with (**d**) associated phasor plot and color coding (pink, long lifetime; green, intermediate lifetime; red, short lifetime). Representative of *n* = 3. (**e**) Consistency in independent phasor populations across multiple measurements. Points, individual biological replicates. Cross, mean of *n* = 3, Error SD. Scale bar, 25 µm.

Having demonstrated that RapidFLIM has an increased capacity for the temporal detection of photons compared to a traditional FLIM system, we explored whether RapidFLIM could be used to investigate the impact of nanoparticle size on cell uptake by correlating changes in the lifetime of particles during uptake. Initially, silica nanoparticles of 30 nm and 100 nm were independently incubated with glioblastoma U87 cells over a 10-h time-course to determine whether particle size influenced uptake and evaluate how lifetimes responded during this process. Representative phasor overlay images of nanoparticle uptake in U87 cells showed that the global lifetime populations of 30 nm particles shifted during uptake, towards a longer intermediate population (Figure 2a–e). Interestingly, this was not observed during uptake of the 100 nm nanoparticles, which showed negligible changes in the populations of the same distinct lifetimes during uptake (Supplementary Figure S1).

### 3.3. RapidFLIM Trajectories of Nanoparticles Identifies Significant Changes in Lifetimes Proximal to Membrane Boundaries 

**Figure 3:**
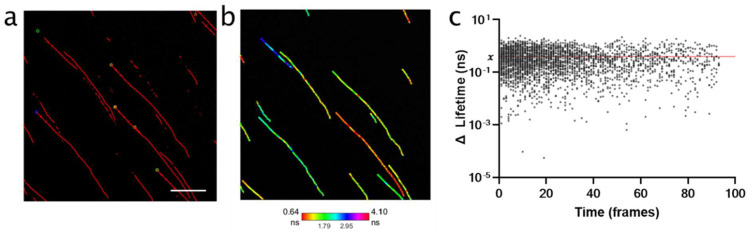
Tracking of 30 nm nanoparticles embedded in 3% agarose to define frame-frame variance (∆ lifetime) within known trajectories. (**a**) Trajectories of 30 nm SiNP defined in DiaTrack, across 100 frames (frame rate 2.62 s), representative of *n* = 2 (with *t* = 3 images per biological replicate). Scale bar, 25 µm. (**b**) Trajectories from (**a**) with pixel specific lifetime overlay. (**c**) The frame-to-frame absolute ∆ lifetime calculated from tracking data (sum of >2200 ∆ lifetimes) which were used to define an average threshold at *x* = 0.39 ns (red line).

Whilst FLIM phasor results demonstrated a global shift from a short to intermediate lifetime of nanoparticles during cell uptake, it was not possible to determine lifetime dynamics on a short imaging timescale (on the order of seconds per frame). We therefore focused on whether the acquisition speed of RapidFLIM could be used to probe changing lifetimes along nanoparticle trajectories during live cell uptake. We first established the background level of lifetime variance (∆ lifetime) by embedding 30 nm nanoparticles in 3% low melt agarose which had an estimated pore size of ~50 nm [29] (Figure 3). Trajectories (representative in Figure 3a,b) were used to define the frame-to-frame lifetime variances, and subsequently define an average variance threshold of 0.39 ns (Figure 3c). 

**Figure 4:**
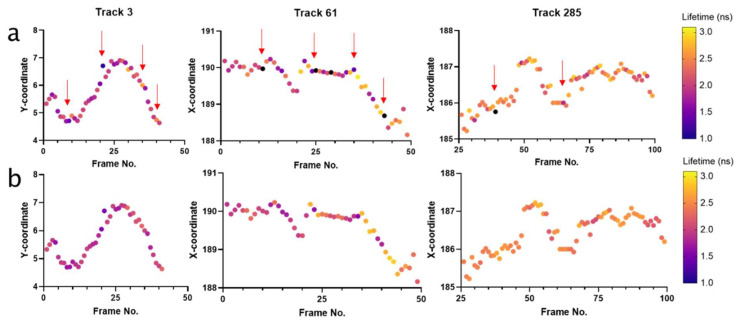
Biological nanoparticle trajectories pre and post smoothing (moving median of three frames). (**a**) Three representative tracks which identified single frame jumps in ∆ lifetime (indicated by red arrows), which influenced the lifetime range of that trajectory. (**b**) Tracks in panel (**a**) post-smoothing.

Thus, across a single trajectory, a lifetime range above the background variation (0.39 ns) would suggest a change in the molecular environment which was influencing the lifetime of the nanoparticle. Prior to applying this threshold to biological data, it was noted that, in some biological tracks, there was a single frame–frame lifetime fluctuation which would be sufficient to meet this threshold. To minimize the likelihood that a significant frame–frame fluctuation was solely responsible for this increase in lifetime range in a biological trajectory (Figure 4a), tracks were first smoothed using a moving median of three frames before the threshold was applied (Figure 4b). The lifetime variance threshold was then applied to the biological datasets, to determine whether the lifetime range of any tracks exceeded the background variance and, therefore, represented a shifting lifetime of potential biological significance. 

**Figure 5:**
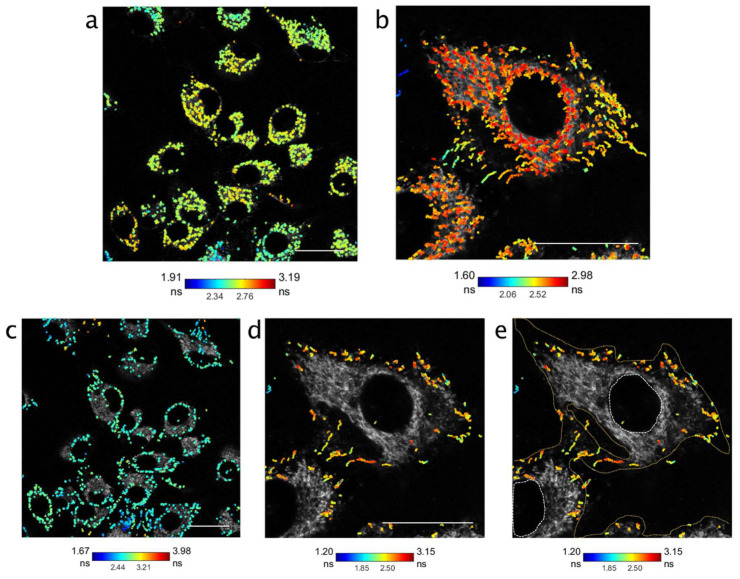
Localization of nanoparticle trajectories during dynamic imaging to identify changing lifetimes over single trajectories (>120 unbinned frames, with sustained photon counts >20 Mcps). Imaged at 24 h post nanoparticle addition, measured using PicoQuant RapidFLIM. (**a**) Tracks where the lifetime range over each trajectory was less than the mean ∆ lifetime (*x* = 0.39 ns) at 24 h appeared to lie within cytoplasmic boundaries. Frame rate 2.62 s, with cumulative trajectories across >150 frames. (**b**) Trajectories in a single cell identifying longer lifetimes within the cytoplasmic compartment. Cumulative trajectories across 120 frames, with frame rate of 0.66 s. (**c**) In contrast, tracks above the mean ∆ lifetime shown to localize near cell boundaries, both internally and externally. (**d**) Nanoparticle tracks in a single cell, above the mean ∆ lifetime with (**e**) demarcation of (**d**) with extracellular and nuclear boundaries (yellow and white dotted lines, respectively). Scale bar in all panels, 25 µm.

Data from time-course imaging at 4 and 6 h post nanoparticle addition identified a holistic increase in the lifetime population at a single trajectory level (Supplementary Figure S2), which agreed with the FLIM data from Figure 2. With the speed of photon detection permitted by RapidFLIM, we investigated nanoparticle lifetime dynamics during cell uptake using a series of unbinned frames (a minimum of 120) at a single timepoint (24 h post addition) which equated to a dynamic dataset captured in less than 10 min (single frame rate of 0.66–2.62 s, and photon counts sustained at >20 Mcps). Intriguingly, when trajectories from these datasets were partitioned between those that had a lifetime range below the mean ∆ lifetime (range < 0.39 ns) versus those with a range greater than 0.39 ns, the localization of tracks appeared cell-compartment specific (Figure 5). Nanoparticle tracks where the lifetime range was below that of the mean variation (<0.39 ns) were shown to lie predominately in intracellular spaces (Figure 5a,b), with an identifiable increase in the overall lifetime (a shift from blue to yellow to red) as nanoparticles moved further into the cytoplasmic compartment (Supplementary Video S1). In contrast, trajectories that had a lifetime range greater than the mean lifetime were predominately located at cellular boundaries (either nuclear or extracellular) (Figure 5c–e).

## 4. Discussion

The study of therapeutic nanoparticles that respond directly to their environment presents an exciting challenge in microscopy. While these particles have attracted great interest, the complexity of their design and release profiles has impeded their translation into clinical use [15,30]. Methodologies that can improve our understanding of how the biophysical properties of the nanoparticles impact their cellular uptake have potential to enrich existing methods for nanoparticle evaluation, to collectively advance nanoparticle research [5,31,32,33,34,35].

We have hereby demonstrated the capacity of a RapidFLIM system to investigate dynamic biological processes including the subcellular localization and uptake of silica nanoparticles in cancer cells. This capitalized on the improved timing electronics of the hybrid detectors used in this system [18], which enabled the accurate detection of fluorescent lifetimes with photon counts at a significantly faster rate than previously permitted by FLIM. 

This speed enabled us to identify single trajectory populations during nanoparticle uptake which showed a correlation between the navigation of nanoparticles across cell boundaries with significant changes in the lifetime range of these tracks. This supports the proposed potential of RapidFLIM for the study of mechanisms and cellular processes which occur at a rapid pace, including vesicle and organelle trafficking [18]. Previous studies of nanoparticle uptake have typically used a combination of microscopy, forced genetic experiments and invasive biological studies to determine the specific mechanism of nanoparticle uptake. For example, one study determined that gold nanoparticles were taken up by HeLa cells via clathrin-mediated endocytosis, using transmission electron microscopy and confocal microscopy, and the blocking of endocytosis using sodium azide [14]. Similarly, clathrin-mediated endocytosis, caveolae-mediated endocytosis and macropinocytosis were shown to be involved in the internalization of polymeric nanoparticles, subject to rigorous endocytic inhibition and microscopy to validate these mechanisms of uptake [13]. Our results demonstrate the ability of RapidFLIM to simultaneously acquire the lifetime and localization of nanoparticles and track these particles during cellular uptake, correlating changes in trajectory lifetimes with key cell boundaries. FLIM has previously been used to study nanoparticle uptake, i.e., by visualizing drug release from drug-loaded nanoparticles [10]. However, this was conducted using global fluorescent lifetime shifts in cells, due primarily to the limitations of FLIM with its slower speed of photon detection [36]. While the current study did not extend to drug-loaded nanoparticles, we hypothesize that RapidFLIM, and the associated analysis pipelines used here [37], could be applied to further investigate mechanisms of nanoparticle uptake and accurately follow intracellular trafficking of nanoparticles and nanoparticle–drug conjugates on a nanosecond timescale. Our study supports the capacity of RapidFLIM to detect and quantify nanosecond shifts in nanoparticle lifetimes during cellular uptake, which has great potential benefit in mechanistic studies of both environmentally responsive and drug-loaded nanoparticles.

RapidFLIM also has broader application to preserve temporal dynamics during the biosensing of metabolic states, such as FAD or NADH cycling [38,39,40]. Given the demonstrated detection of RapidFLIM here to be greater than traditional FLIM, the imaging of these processes using RapidFLIM has the potential to increase the cellular sensitivity and localization of FAD and NADH detection, such as by harnessing existing nanoparticle or polymer-based detection systems [41,42] and endowing these with a higher temporal resolution.

## 5. Conclusions

To the authors’ knowledge, this study marks the first application of RapidFLIM to study how single lifetime trajectories can be used to correlate the biophysical characteristics of nanoparticles with their cellular environment, during live cell uptake. We have suggested parameters for consideration when using a RapidFLIM system and demonstrated the capacity of this technology to simultaneously capture the fluorescent lifetimes, subcellular localization and uptake of nanoparticles in live cells at a single-cell resolution, and on a nanosecond timescale. We believe that this technology and associated analyses have the potential to streamline mechanistic studies of nanoparticle delivery, thus advancing our understanding of nanoparticle uptake in cells and improving their translation as nanomedicines for future cancer therapy.

## Data Availability

Data available from authors on request. Further, to facilitate use of custom tracking analysis described herein, a data analysis package has been made available via GitHub, with the capacity for future updates and improvements: Cells Fluorescence Lifetime Tracking Analysis. Available online: https://github.com/ElvPan/Cells-Fluorescence-Lifetime-Tracking-Analysis (accessed on 9 January 2022). DiaTrack software used for initial particle tracking is also publicly available. DiaTrack Particle Tracking Software. Available online: https://download.cnet.com/DiaTrack/3000-2054_4-76212704.html (accessed on 9 January 2022).

## Supplementary Materials

The following supporting information can be downloaded at: https://www.mdpi.com/article/10.3390/cells11040642/s1, Figure S1: Time-lapse imaging of 100 nm silica nanoparticles (SiNP) uptake and lifetime in U87 glioblastoma cells; Figure S2: Localization of nanoparticle trajectories where the lifetime range was greater than the mean ∆ lifetime; Video S1: Frame by frame video of nanoparticle uptake 24 h post addition with trajectories below the mean ∆ lifetime.
