# Supplementary material for: Application of Rapid Fluorescence Lifetime Imaging Microscopy (RapidFLIM) to Examine Dynamics of Nanoparticle Uptake in Live Cells
Source: Cells. 2022 Feb 12;11(4):642. doi: 10.3390/cells11040642 (PMC8870300; doi:10.3390/cells11040642)
Supplement: Supplementary file 1 [file cells-11-00642-s001.zip › cells-1570540-supplementary.pdf]

Application of Rapid Fluorescence Lifetime Imaging Microscopy (RapidFLIM) to examine dynamics of nanoparticle uptake in live cells

Aria Ahmed-Cox,[1,2,3] *Alexander M. Macmillan,*[4] *Elvis Pandzic,*[4] *Renee M. Whan,*[4] *and Maria Kavallaris*[1,2,3]*

[1] Children's Cancer Institute, Lowy Cancer Research Center, UNSW Sydney, Randwick, NSW 2031, Australia

[2] ARC Center of Excellence in Convergent Bio-Nano Science and Technology, Australian Center for NanoMedicine, UNSW Sydney, Sydney, NSW 2031, Australia

[3] School of Women and Children's Health, Faculty of Medicine and Health, UNSW Sydney, Sydney, NSW 2031, Australia

[4] Katharina Gaus Light Microscopy Facility, Mark Wainwright Analytical Center, UNSW Sydney, Sydney NSW, 2031, Australia

* Corresponding author: Maria Kavallaris m.kavallaris@ccia.unsw.edu.au

Supplementary Material

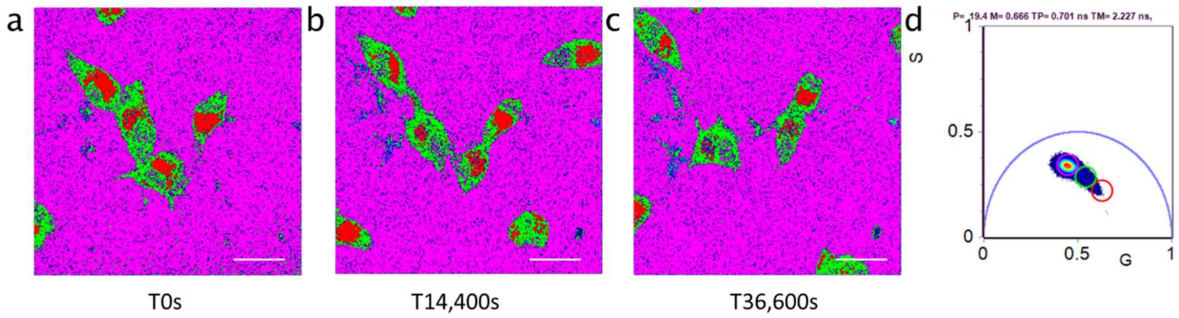

Supplementary Figure S1 Time-lapse imaging of 100 nm silica nanoparticles (SiNP) uptake in U87 glioblastoma cells captured over 10 h (36,600 s) following SiNP addition. Representative phasor overlay for 100 nm SiNP at **(a)** time 0, **(b)** 4 h and **(c)** 10 h following SiNP addition, with **(d)** associated phasor plot and color coding (pink, long lifetime; green, intermediate lifetime; red, short lifetime). Representative of $n = 2$. Scale bar, 25 µm.

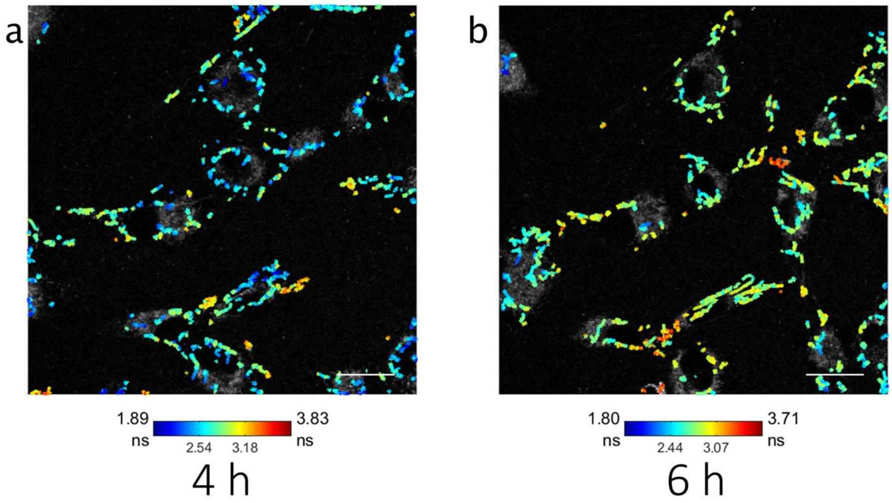

Supplementary Figure S2 Localization of nanoparticle trajectories where the lifetime range was greater than the mean Δ lifetime (x = 0.39 ns), measured using PicoQuant RapidFLIM. Trajectories at **(a)** 4 h and **(b)** 6 h post nanoparticle addition were overlayed on the original image. Scale bar in all panels, 25 µm.

Supplementary Video S1 Frame by frame video of nanoparticle uptake 24 h post addition with trajectories only below the mean Δ lifetime (x = 0.39 ns), measured using PicoQuant RapidFLIM. This video can be accessed via FigShare:

Ahmed-Cox (2022) RapidFLIM Supplementary Figure S1. Available online: https://doi.org/10.6084/m9.figshare.18094148.v1 (accessed on 12/02/2022).
